# Osteocalcin and GPR158: linking bone and brain function

**DOI:** 10.3389/fcell.2025.1564751

**Published:** 2025-04-23

**Authors:** Jingjing Li, Shujie Lou, Xuepeng Bian

**Affiliations:** ^1^ Physical Education College, Shanghai University, Shanghai, China; ^2^ School of Exercise and Health, Shanghai University of Sport, Shanghai, China; ^3^ Department of Rehabilitation, School of International Medical Technology, Shanghai Sanda University, Shanghai, China

**Keywords:** osteocalcin, GPR158, neurodegenerative diseases, cellular activity, synaptic plasticity, metabolism

## Abstract

Osteocalcin (OCN), a small protein secreted by osteoblasts, has attracted significant attention for its role as an endocrine factor in regulating the central nervous system (CNS) via the bone-brain axis. As a critical receptor for OCN, G protein-coupled receptor 158 (GPR158) facilitates the proliferation, differentiation, and survival of neural cells while directly influencing neurons' structural and functional plasticity, thereby modulating cognitive function. Additionally, GPR158 is involved in cellular energy metabolism and interacts with proteins such as regulators of G protein signaling 7 (RGS7), broadening the understanding of OCN’s impact on neural activity. Notably, GPR158 displays region- and cell type-specific bidirectional effects under certain pathological conditions, such as tumor development and mood regulation, adding complexity to its mechanisms of action. Although the precise biological mechanisms underlying the OCN/GPR158 signaling pathway remain incompletely understood, its association with neurodegenerative diseases (NDs), including Alzheimer’s disease (AD) and Parkinson’s disease (PD), is becoming increasingly evident. Thus, a systematic summary of OCN/GPR158 in CNS regulation and NDs will deepen understanding of its role in brain function and support the development of new therapeutic targets and strategies.

## 1 Introduction

With the expanding recognition of interorgan crosstalk, such as the liver-brain, muscle-brain, and gut-brain axes, research on biomolecules influencing neurodegenerative diseases (NDs) has transcended traditional boundaries. However, interactions between peripheral organs and the central nervous system (CNS), mainly via the bone-brain axis, remain comparatively underexplored. Traditionally regarded primarily as structural components facilitating support and motor, bones have recently been recognized for their broader physiological roles. Osteocalcin (OCN), a non-collagen matrix protein secreted by osteoblasts, is a critical marker of bone formation and metabolism and functions as an endocrine hormone. Upon entering the circulatory system, OCN modulates peripheral energy metabolism, insulin sensitivity, and muscle function (Ferron et al., 2008; Zhao et al., 2023; Correa Pinto Junior et al., 2024). Additionally, its emerging roles in cognition and emotion have attracted increasing scholarly attention (Oury et al., 2013).

OCN acts through receptors such as GPRC6A, GPR37, and GPR158. The peripheral effects of OCN, including the regulation of glycolipid metabolism and insulin secretion, are primarily mediated by the GPRC6A, which is exclusively expressed in peripheral tissues. In contrast, GPR37 and GPR158 are predominantly expressed in the CNS and are likely to mediate the effects of OCN on brain function. Although GPR37 supports neuronal migration, glial cell differentiation, and myelination (Bian et al., 2024), its involvement in OCN-mediated synaptic regulation appears limited. GPR37 functions primarily through glial cells and lacks direct regulatory capacity over synaptic plasticity and higher-order neural processes such as emotion and cognition (Bian et al., 2024). Conversely, GPR158 exhibits neuron-specific expression in key brain regions, including the cerebral cortex, hippocampus, and hypothalamus, and is directly involved in modulating synaptic structure and functional plasticity. It has been implicated as a central mediator in neuropsychiatric conditions such as stress, depression, and cognitive impairment. Recent studies further identify GPR158 as a critical receptor mediating OCN’s regulation of central energy metabolism, a function in which GPR37 plays only a limited role (Table 1). Moreover, GPR37 activation has been associated with enhanced intracellular stress responses (Imai et al., 2001; Marazziti et al., 2009), which contrasts with the protective effects of OCN against oxidative stress (Wu et al., 2021). These functional divergences suggest that OCN’s actions in the brain are not entirely dependent on GPR37, and that GPR158 may play a compensatory or complementary role in brain regions and processes beyond the scope of GPR37.

**TABLE 1:** Summary of OCN ligands, distribution, and major research areas.

Receptors	Primary expression	Major research areas
GPR158	Central: neuron-specific expression in the cerebral cortex, hippocampus, and hypothalamus Peripheral: adrenal gland, pancreas, trabecular meshwork cells	Neural plasticity and cognition (Rivagorda et al., 2025), stress response and depression (Sutton et al., 2018), endocrine and metabolic regulation (Fu et al., 2022; Lin et al., 2022), tumor progression (Fu et al., 2022; Sakellakis, 2022)
GPR37	Central: dopaminergic neurons, oligodendrocytes, astrocytes Peripheral: macrophages, smooth muscle cells, cardiomyocytes, alveolar epithelial cells	Oligodendrocyte maturation and myelination (Qian et al., 2021), Parkinson’s disease (Marazziti et al., 2004; Zhang et al., 2020b), neuronal function and survival (Owino et al., 2021), inflammation (Bolinger et al., 2023), tumor progression (Xie et al., 2022; Zhou et al., 2024)
GPRC6A	Peripheral: skeletal muscle, immune cells, Leydig cells, anterior pituitary, osteoblasts, pancreatic β-cells, liver, adipose tissue	Metabolic regulation, bone-muscle axis (Sun et al., 2022), male reproduction (Karsenty and Oury, 2014; Taib and Jayusman, 2024), inflammation (Clemmensen et al., 2014), tumor progression (Pi et al., 2018)

Therefore, targeting GPR158 may provide novel insights into how OCN regulates brain function and offer new directions for investigating the bone–brain axis in NDs.

## 2 Physiological functions of OCN in the bone and brain

### 2.1 OCN and bone

OCN is one of the most abundant proteins in the bone matrix and exists in two distinct forms: carboxylated osteocalcin (cOCN) and undercarboxylated osteocalcin (ucOCN). cOCN primarily contributes to bone mineralization, whereas ucOCN exerts endocrine functions and regulates various physiological processes, including bone metabolism.

#### 2.1.1 Bone mineralization and structural adjustment

Bone formation is a highly dynamic physiological process that progresses through four sequential stages: pre-osteogenesis, matrix synthesis, mineralization, and maturation. During the transition from pre-osteogenesis to matrix synthesis, the expression of osteocalcin OCN gradually increases from a low baseline. Initially, OCN facilitates the differentiation of mesenchymal stem cells and promotes the maturation of osteoblasts (Moriishi et al., 2020). OCN is progressively incorporated into the newly synthesized extracellular matrix as the bone matrix forms, further enhancing matrix deposition and osteoblast maturation (Hosseini et al., 2019). Bone mineralization represents a critical phase that determines bone quality and mechanical strength. Although non-collagenous proteins (NCPs) are present in smaller quantities than collagen within the bone matrix, they play indispensable roles in regulating calcium ion binding, hydroxyapatite nucleation, and crystal growth. Among these, the small integrin-binding ligand N-linked glycoprotein (SIBLING) family—including dentin matrix protein 1 (DMP1), bone sialoprotein (BSP), and osteopontin (OPN)—exerts fine control over mineral deposition via specialized functional domains (Silvent et al., 2013; Vijaykumar et al., 2020). Within this regulatory network, OCN is a critical mediator linking the organic matrix to mineral components. At this stage, OCN is extensively distributed throughout the mineralized matrix and reaches its peak expression level (Xu et al., 2023). Studies have demonstrated that the molecular structure of cOCN contains γ-carboxyglutamic acid (Gla) residues, which exhibit a high binding affinity for calcium ions. Upon binding to Ca^2+^, OCN functions as a mineralization inducer by promoting the deposition of phosphate PO_4_^3-^, ultimately facilitating hydroxyapatite formation (Tavakol et al., 2024). This process enhances bone matrix mineralization and contributes to increased bone density.

In addition to its role in mineral deposition, OCN is crucial in optimizing the crystalline organization of bone minerals. By ensuring that mineral particles are systematically aligned along collagen fibers, OCN significantly enhances the mechanical strength of bone (Manolagas, 2020). Despite the presence of mineral deposits in bone following OCN gene knockout, the disorganized arrangement of mineral crystals results in a marked reduction in bone strength (Xu et al., 2023), highlighting the essential role of OCN in regulating bone structure and maintaining its biomechanical properties.

#### 2.1.2 Bone remodeling

Bone remodeling is a dynamic equilibrium process that involves the coordinated regulation of bone formation and resorption. The functions of osteocalcin OCN are multifaceted. First, OCN promotes bone formation by stimulating osteoblasts to synthesize bone matrix proteins. Second, OCN influences the differentiation and activity of osteoclasts and regulates bone resorption through its interaction with specific receptors, such as GPRC6A (Wang H. et al., 2021). Additionally, OCN modulates the secretion of key regulatory factors, including transforming growth factor beta, fibroblast growth factor 23, and osteopontin, by osteoblasts. Through these mechanisms, OCN indirectly influences osteoclast activity and contributes to the regulation of bone resorption (Lee et al., 2007).

OCN not only directly regulates the activity of bone cells but also interacts with other hormones through an intricate endocrine network to collectively modulate bone metabolism. Among these hormones, testosterone is closely associated with OCN function. Studies have demonstrated a significant positive correlation between circulating OCN levels and serum testosterone concentrations (Kanazawa et al., 2013; Zhong et al., 2016). OCN enhances testosterone synthesis by upregulating key steroidogenic enzymes, including cytochrome P450 family 11 subfamily A member 1 (CYP11A1, CYP17A1), and hydroxy-delta-5-steroid dehydrogenase three beta-and steroid delta-isomerase 1 (HSD3β1 and HSD3β6), in a cyclic AMP response element-binding protein (CREB)-dependent manner. This regulatory mechanism is mediated through the binding of OCN to the GPRC6A in testicular interstitial cells, leading to a significant increase in testosterone secretion (Bharath Kumar et al., 2024) and promoting germ cell survival (Oury et al., 2011; Oury et al., 2015; Jawich et al., 2022).

Comparative studies in OCN-deficient male mice have revealed decreased sperm counts and lower circulating testosterone levels, resulting in reduced reproductive capacity (Li and Li, 2014). In addition to its role in reproductive function, testosterone exerts anabolic effects on bone metabolism by stimulating osteoblast activity, promoting bone matrix synthesis, and inhibiting osteoclast function, thereby reducing the risk of bone loss. Furthermore, testosterone undergoes aromatization to estrogen, a process that further enhances bone mineral density (Kanazawa et al., 2013; Zhong et al., 2016).

#### 2.1.3 Osteocytic feedback regulation of OCN secretion by osteoblasts

During bone formation, portions of osteoblasts become embedded within the self-secreted bone matrix and gradually differentiate into osteocytes, thereby establishing the osteocyte network within bone tissue. Osteocytes exert regulatory feedback on osteoblast activity through the secretion of sclerostin, which binds to low-density lipoprotein receptor-related proteins 5 and 6 (LRP5/6) receptors on osteoblast membranes (Delgado-Calle and Bellido, 2022). This interaction inhibits Wnt/β-catenin signaling and downregulates the expression of OCN. Conversely, sclerostin inhibition enhances Wnt/β-catenin signaling, increasing bone formation and elevated OCN expression (Hu et al., 2024).

Osteocytes also play a central role in the regulation of osteoclastogenesis and bone resorption via the secretion of receptor activator of nuclear factor-κB ligand (RANKL) and osteoprotegerin (OPG) (Delgado-Calle and Bellido, 2022). RANKL binds to its receptor RANK on osteoclast precursors, inducing their differentiation into mature osteoclasts and promoting bone matrix resorption. The degradation of the bone matrix releases OCN into circulation, where it functions in various endocrine and paracrine signaling pathways (Wang J. S. et al., 2021). Additionally, moderate bone resorption facilitates the release of growth factors sequestered in the matrix, stimulating new bone formation and supporting the redeposition of OCN.

Both osteoblasts and osteocytes are capable of secreting fibroblast growth factor 23 (FGF23), which negatively regulates OCN synthesis indirectly by suppressing circulating levels of 1,25-dihydroxyvitamin D_3_ [1,25(OH)_2_D_3_] (Zhang et al., 1997). Chronically elevated FGF23 levels, as observed in disorders such as tumor-induced osteomalacia and X-linked hypophosphatemic rickets, can lead to hypophosphatemia and impaired bone mineralization (Dallas et al., 2013). Under these conditions, OCN deposition within the bone matrix is diminished, potentially compromising its functional integration into the mineralized structure.

### 2.2 OCN and brain function

OCN circulates through the bloodstream and reaches various tissues and organs, exerting various endocrine hormone-like effects. Beyond its well-established role in bone metabolism, OCN is critical in regulating brain function.

#### 2.2.1 Cognitive function

A significant positive correlation has been observed between OCN levels and cognitive function. Reduced OCN concentrations in cerebrospinal fluid have been documented in various neurodegenerative disorders, including Alzheimer’s disease (AD) and Parkinson’s disease (PD) (Hou et al., 2021; Liu et al., 2023). Mice deficient in OCN exhibited impaired spatial learning and memory dependent on the hippocampus (Oury et al., 2013).

Furthermore, OCN supplementation has enhanced cognitive function by reducing amyloid-beta (Aβ) accumulation and gliosis in the hippocampus and cortex. Additionally, OCN increases monoamine neurotransmitters, brain-derived neurotrophic factor (BDNF), and other synaptic plasticity-associated proteins, thereby promoting neuronal plasticity (Shan et al., 2023).

Notably, OCN regulates brain function throughout the life cycle. OCN crosses the placenta during fetal development to facilitate nervous system development, and maternal OCN deficiency has been linked to abnormal brain development (Oury et al., 2013). In aging populations, the age-related decline in OCN levels has been associated with cognitive deterioration, while exogenous OCN supplementation has been shown to reverse age-related cognitive decline (Oury et al., 2013; Correa Pinto Junior et al., 2024).

#### 2.2.2 Mood and stress response

Beyond its role in cognitive function, OCN is integral to mood regulation. OCN stimulates the synthesis of monoamine neurotransmitters—including serotonin (5-HT), dopamine (DA), and norepinephrine (NE)—thereby directly influencing mood states (Berger et al., 2019). OCN-deficient mice exhibit anxiety-like and depression-like behaviors, which are alleviated by exogenous OCN supplementation (Oury et al., 2013).

Furthermore, OCN is essential for acute stress responses. Notably, acute stress reactions can occur independently of adrenal gland function or even in cases of adrenal insufficiency, and these responses are closely associated with a rapid surge in circulating OCN levels (Berger et al., 2019). Research indicates that exposure to stressors results in increased OCN levels within minutes. This response is directly linked to bone activity, as osteoblasts facilitate the release of bioactive OCN via glutamate uptake (Berger et al., 2019). Unlike conventional stress responses, this mechanism operates independently of classical stress hormone pathways, such as corticosterone and catecholamines (Berger et al., 2019). These findings underscore the pivotal role of OCN in acute stress adaptation.

**FIGURE 1:**
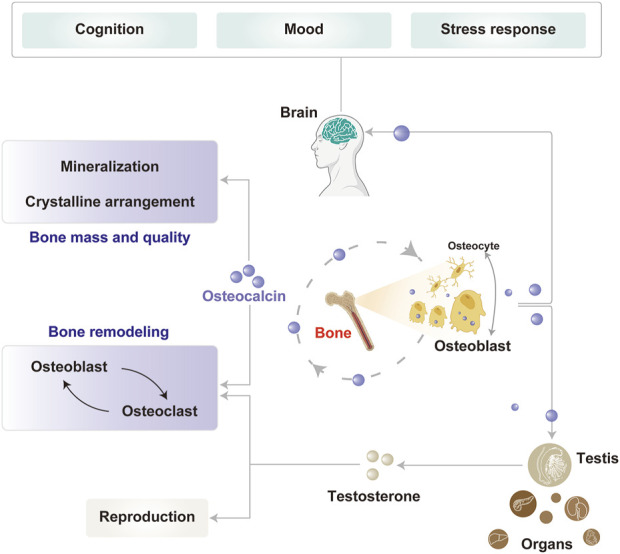
Physiological functions of OCN in the bone and brain. OCN, primarily secreted by osteoblasts, plays key roles in bone mineralization, remodeling, and reproduction. Its expression is modulated in part by osteocytes through feedback regulation. Circulating OCN also acts on the brain to influence cognition, mood, and stress responses.

In conclusion, OCN is essential for maintaining bone health by contributing to bone mineralization, structural remodeling, and regulating bone metabolism by balancing bone formation and resorption. Beyond its bone functions, OCN also plays a pivotal role in brain function, primarily influencing cognition, mood regulation, and stress response (Figure 1).

## 3 Multiple roles of OCN/GPR158 in the CNS

Reduced expression of osteoblast markers, including OCN and osteopontin, has been observed in spinal muscular atrophy (SMA) (Shanmugarajan et al., 2009), while GPR158 knockout impairs novelty preference in autism spectrum disorders (ASD) (Wei et al., 2024). Overexpression of OCN elevates hippocampal BDNF levels, enhancing spatial learning and memory via GPR158 while also reducing anxiety, Aβ accumulation, and glial proliferation in AD (Sun et al., 2021; Shan et al., 2023). These findings establish a connection between OCN/GPR158 and bone health with NDs through their mediating roles in spatial memory and emotional regulation (Cetereisi et al., 2019). This relationship underscores the importance of further investigating the physiological mechanisms underlying the function of OCN/GPR158 in the CNS.

### 3.1 OCN/GPR158 regulates neuronal proliferation and cell survival

Maternal OCN crosses the placenta during pregnancy, preventing neuronal apoptosis before the embryo produces OCN autonomously, thereby supporting fetal brain development (Oury et al., 2013). Additionally, OCN at various concentrations significantly enhances the proliferation of PC12 cells, promotes neurite outgrowth, and facilitates nerve growth factor (NGF)-induced cell differentiation (Ando et al., 2021). GPR158 may mediate the promotive effects of OCN, as its knockdown suppresses the cell cycle regulator Cyclin D1 (Patel et al., 2013). Furthermore, the eighth helix of GPR158 is an α-helical region containing a nuclear localization signal (NLS). Mutations in this region result in the loss of GPR158-mediated pro-proliferative effects (Patel et al., 2013), indicating that nuclear localization of GPR158 is critical for its pro-proliferative function. Additionally, GPR158 negatively regulates genes associated with the unfolded protein response (UPR) during endoplasmic reticulum stress (ERS), including heat shock protein family A (Hsp70) member 5 (HSPA5), X-box binding protein 1 (XBP1), activating transcription factor 4 (ATF4) and C/EBP homologous protein (CHOP) (Suarez et al., 2023). The alleviation of ERS concurrently contributes to the protection of cell survival (Patel et al., 2013; Itakura et al., 2019; Suarez et al., 2023).

However, the role of GPR158 in brain tumor cells appears to be multifaced. On the one hand, the overexpression of GPR158 in brain tumor stem-like cells (BTSCs) has inhibited cell proliferation and migration while promoting cell differentiation and apoptosis. Conversely, GPR158 downregulation, such as by miR-449a, directly targets its 3′UTR, promoting the proliferation, migration, and self-renewal capacity of BTSCs while inhibiting their differentiation and apoptosis (Li et al., 2018). These effects may be associated with GPR158-mediated activation of the tumor protein 53 (TP53), a transcription factor that responds to cellular stress and halts cell replication by maintaining the cell cycle at the G1/S checkpoints (Suarez et al., 2023). On the other hand, in low-grade neurodifferentiated gliomas and neuroendocrine tumors, such as pheochromocytoma and paraganglioma, GPR158 is highly expressed (Wei et al., 2024). Additionally, GPR158 promotes tumor cell proliferation and angiogenesis and may be negatively regulated by miR-613 (Wang et al., 2022).

**FIGURE 2:**
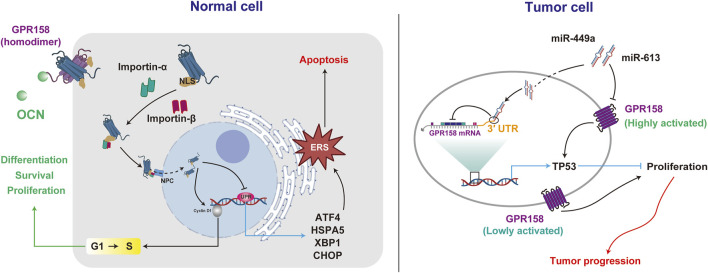
GPR158 exhibits different roles in regulating cellular activities in normal and tumor cells. GPR158 facilitates cell proliferation, differentiation, and survival in normal cells by modulating the G1/S checkpoint. Additionally, it suppresses genes associated with the unfolded protein response (UPR). In tumor cells, GPR158 demonstrates dual effects on proliferation, exhibiting context-dependent behavior under high or low activation states.

The diverse effects of GPR158 in tumor cells may stem from its spatiotemporal expression patterns and expression levels. Studies suggest that GPR158 overexpression differentially modulates UPR marker expression depending on the dosage. Notably, transient transfection of GPR158 promotes proliferation in prostate cancer cells. However, in a lentiviral stable transfection model, low doses of GPR158 enhance cell proliferation, whereas high doses exert an inhibitory effect. The bidirectional and complex nature of OCN/GPR158 may provide novel insights into preventing and treating NDs (Figure 2).

### 3.2 OCN/GPR158 promotes synaptic plasticity

Synaptic plasticity is the ability of synapses to undergo structural and functional modifications, forming the biological basis for learning, adaptation, and recovery in the CNS (Martin et al., 2000; Bin Ibrahim et al., 2022). This process includes both short-term plasticity, such as paired-pulse facilitation driven by presynaptic neurotransmitter release, and long-term plasticity, exemplified by long-term potentiation (LTP) and long-term depression (LTD), which entail alterations of postsynaptic receptors. Notably, OCN/GPR158 signaling is critical in modulating synaptic plasticity. Specifically, OCN supplementation enhances the action potentials (APs) frequency of cornu ammonis 3 (CA3) pyramidal neurons and promotes LTP in the mossy fiber (MF)-CA3, leading to improved hippocampal-dependent memory. The generation of APs originates from the release of neurotransmitters. OCN knockout mice show reduced NE, 5-HT, and DA levels, increased GABA, and exhibit anxiety, depressive-like behavior, and cognitive impairments. OCN supplementation enhances key neurotransmitter-synthesizing enzymes, including Glutamate Decarboxylase 1/2 (GAD1/2), Tryptophan Hydroxylase 2 (TPH2), and Tyrosine Hydroxylase (TH) (Oury et al., 2013). The plasticity changes driven by GPR158 modulation align with those observed for OCN. Activation of GPR158 markedly enhances APs frequency and reduces the threshold current necessary to elicit the initial APs (Laboute et al., 2023). In GPR158 knockout models, these enhancements are abolished, along with a marked reduction in synaptic structure and complexity in hippocampal CA1 and CA3 neurons (Condomitti et al., 2018).

Regional differences in synaptic plasticity regulation by OCN/GPR158 are evident. In GPR158−/− mice, hippocampal CA1 pyramidal neurons predominantly exhibit weakened postsynaptic functions characterized by reduced postsynaptic currents. In contrast, the CA3 region demonstrates impairments in both presynaptic and postsynaptic structures and functions, including reduced paired-pulse facilitation (PPF), shortened synaptic active zone (AZ), and postsynaptic density (PSD) lengths, as well as decreased frequency and amplitude of spontaneous excitatory postsynaptic currents (sEPSCs) (Condomitti et al., 2018). Furthermore, GPR158 demonstrates a distinct expression pattern at the cellular level, being enriched in excitatory neurons while limited in inhibitory interneurons (Chang et al., 2023). This differential expression pattern serves as the structural basis for the varying effects of GPR158 on excitatory and inhibitory neurons. In the mPFC of GPR158−/− mice, a reduction in synaptic vesicles at excitatory synapses was observed, accompanied by decreased expression and phosphorylation of GluN2B, resulting in a marked impairment of synaptic transmission. Notably, inhibitory synapses remained unaffected (Wei et al., 2024).

GPR158 modulates synaptic plasticity through multiple signaling pathways. Its activation downregulates the Kv7.2/KCNQ potassium channel via PKA and ERK pathways, decreasing M current amplitude and increasing the excitability of medium spiny neurons (MSNs) (Aceto et al., 2024). OCN binds to GPR158, activating the IP3R and retinoblastoma-associated protein 48 (RbAp48) pathways to upregulate BDNF expression, enhance BDNF-enriched vesicle transport, and increase action potential frequency and LTP in the MF pathway, thereby improving cognitive deficits in aged mice (Khrimian et al., 2017; Kosmidis et al., 2018). Transcriptomic data from the mouse cerebral cortex reveal that GPR158 influences the expression of synaptosome-associated protein 25 (Snap25), a key component of the soluble N-ethylmaleimide-sensitive factor attachment protein receptors (SNARE) complex. Snap25 plays a critical role in coordinating calcium signaling to regulate exocytosis-endocytosis coupling. Inhibition of the Gβγ subunit signaling pathway, upon which Snap25 depends, disrupts the GPCR (G protein-coupled receptor)-SNARE interaction, leading to suppressed glutamatergic neurotransmitter release, impaired LTP, and deficits in learning and memory, accompanied by other behavioral abnormalities (Manz et al., 2023).

**FIGURE 3:**
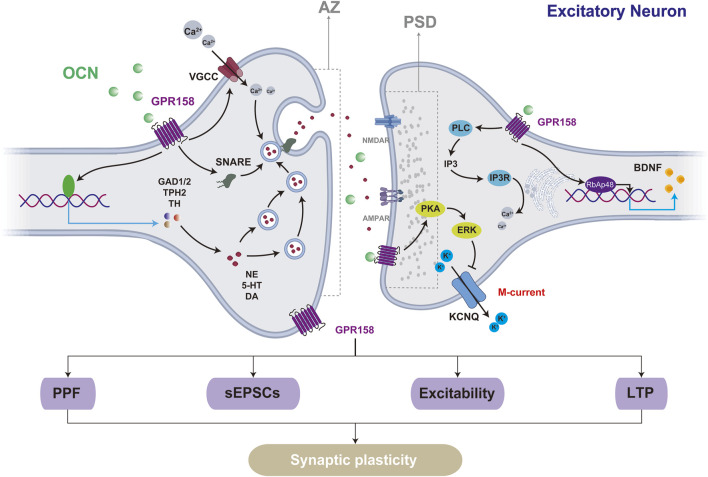
GPR158 influences synaptic plasticity in excitatory neurons through multiple mechanisms. Structurally, the activation of GPR158 enhances the lengths of both the AZ and the PSD. Functionally, GPR158 activation promotes short-term plasticity, such as PPF driven by presynaptic neurotransmitter release, and long-term plasticity, as demonstrated by LTP. The underlying mechanisms may involve GPR158-mediated inhibition of the M-current and increased expression of BDNF. VGCC, Voltage-gated calcium channels; KCNQ, Potassium voltage-gated channel, subfamily Q.

Contrary to the prevailing view that GPR158 promotes synaptic plasticity, GPR158 knockout enhances glutamatergic neuron plasticity in the mouse mPFC, increasing BDNF expression, dendritic spine density, sEPSCs frequency, and AMPA/NMDA ratio, leading to antidepressant and anti-stress behaviors (Sutton et al., 2018). Elevated baseline levels of GPR158 observed in the stress model may partly explain the contrasting results, as another study identified GPR158 as promoting cell proliferation at low concentrations while exerting inhibitory effects at higher concentrations (Suarez et al., 2023). Additionally, while GPR158 knockout reduced overall synaptic plasticity in the hippocampus, dendritic spine density in the apical stratum lucidum of CA3 increased by 37% compared to wild-type (WT) mice (Condomitti et al., 2018). These findings highlight the complex and context-dependent role of GPR158 in synaptic plasticity, emphasizing the need for analyses tailored to specific cell types, tissue regions, and disease models (Figure 3).

### 3.3 OCN/GPR158 influences central glucose metabolism to ameliorate NDs

Beyond its effects on neuronal activity, maternal OCN deficiency disrupts gene expression across multiple tissues and organs in offspring, impairing the development of pancreatic islets, testes, and other organs. These disruptions result in progressive metabolic abnormalities, including impaired insulin secretion, dysregulated glucose metabolism, and altered hepatic gluconeogenesis (Ferron et al., 2008; Ferron et al., 2012; Zhang X. L. et al., 2020; Correa Pinto Junior et al., 2024; Paracha et al., 2024). Disruption of peripheral glucose metabolism significantly impacts CNS function (Guo et al., 2020). Metabolomic analyses have revealed substantial impairments in hippocampal glucose metabolism in diabetic rats, characterized by reduced aerobic oxidation and increased reliance on glycolysis (Li et al., 2019a). These metabolic disturbances are closely associated with decreased expression of proteins critical for synaptic plasticity, alongside deficits in working memory (Li et al., 2019b). Notably, these cognitive impairments coincide with reduced serum levels of OCN (Zhao et al., 2024).

In NDs such as AD, PD, and Huntington’s disease (HD), reduced OCN levels are frequently observed, often accompanied by widespread disruptions in CNS glucose metabolism. These alterations in glucose metabolism across multiple brain regions contribute to the accumulation of Aβ and tau proteins, abnormal distribution of alpha-synuclein, and motor deficits (Duan et al., 2003; Patassini et al., 2016; Scholefield et al., 2023; Shan et al., 2023). These findings suggest that OCN plays a critical role in modulating cognition associated with aging and NDs, potentially via its regulation of glucose metabolism. OCN supplementation dose-dependently improves metabolic and diabetes-related cognitive impairments (Zhao et al., 2024). The cognitive benefits of OCN are mediated through its regulation of the insulin signaling pathway, particularly the IRS/PI3K/Akt pathway (Dewanjee et al., 2022). Akt inhibition partially abolishes OCN’s protective effects on cognitive deficits, underscoring OCN’s critical role in regulating insulin signaling to mediate NDs (Zhao et al., 2024).

The interaction between OCN/GPR158 and cell metabolism occurs during the progression of NDs. OCN regulates circulating fasting glucose and total cholesterol levels, indirectly protecting against AD (Guo et al., 2024). Additionally, GPR158 enhances glial aerobic glycolysis, reduces Aβ accumulation, and directly improves cognitive function in AD (Shan et al., 2023). Conversely, chronic hyperglycemia can induce upregulation of the DNA-modifying enzymes (Dnmt1/3b) in the rat hippocampus, which inhibits the expression of GPR158 through epigenetic mechanisms such as methylation (Patricia da Silva et al., 2023). This alteration disrupts the bone-brain axis interactions, adversely affecting cognitive function.

### 3.4 Protein interaction network of OCN/GPR158

GPR158 facilitates presynaptic differentiation in CA3 pyramidal neurons through its interaction with heparan sulfate proteoglycans (HSPGs) and the coreceptor leukocyte common antigen-related (LAR) family receptors (Kamimura and Maeda, 2021). Unlike the canonical structure of GPCRs, GPR158 predominantly forms a dimer stabilized by interactions with phospholipids and cholesterol molecules. Its N-terminal region contains a distinctive Cache domain and a cysteine-rich region (Laboute et al., 2023), which endows the receptor with diverse ligand-binding capabilities and enhanced structural stability.

Though relatively short, the C-terminal region of GPR158 contains a CT-CC domain that interacts with the Regulator of G-protein Signaling 7 (RGS7)-Gβ5 complex (Laboute et al., 2023). RGS7 is broadly expressed in neurons across multiple brain regions, including the cerebral cortex, hippocampus, thalamus, basal ganglia, and cerebellum, and serves as a key modulator of GPCR signaling in the nervous system (Tayou et al., 2016; Jeong et al., 2021). As a G protein regulatory protein, RGS7 negatively regulates GPCR signaling by accelerating the GTP hydrolysis of Gi/o-class G proteins, thereby promoting their inactivation (Patil et al., 2022). Upon complex formation with RGS7-Gβ5, GPR158 translocates from the cytoplasm to the cell membrane, enabling its function in signal recognition (Orlandi et al., 2015). Under stress conditions, GPR158 enhances GTPase activity by binding to the RGS7 complex, thereby establishing a negative feedback pathway that modulates mPFC neuronal activity (Darira and Sutton, 2022). GPR158 has also been identified as a membrane anchor for the RGS7-Gβ5 complex, facilitating its stabilization and localization at the neuronal membrane, enhancing RGS7’s regulatory efficiency on GPCR signaling (Patil et al., 2022).

RbAp48 is a pivotal regulator of chromatin organization and gene expression in the hippocampus, with its elevated expression levels strongly associated with improved cognitive performance. It has been recognized as a critical downstream effector of GPR158. Perturbations in the OCN/GPR158 signaling pathway lead to a significant reduction in RbAp48. The interaction between RbAp48 and GPR158 is fundamental for maintaining cognitive integrity, as hippocampal inhibition of RbAp48 negates the cognitive benefits mediated by OCN, resulting in pronounced deficits in discriminative memory (Kosmidis et al., 2018).

Analysis of AD samples across Braak stages reveals significant downregulation of GPR158 in the cerebral cortex, with an inverse correlation between GPR158 levels and β-secretase activity. β-secretase is a key enzyme in the amyloid precursor protein degradation pathway that generates Aβ, the primary component of amyloid plaques (Zhu et al., 2020). In PD, the pathological aggregation of α-synuclein from its monomeric form into fibrils disrupts synaptic transmission and represents a hallmark of the disease (Ruiperez et al., 2010). GPR158 suppresses α-synuclein fibril formation by interacting with high mobility group box-1 protein (HMGB1) (Mallah et al., 2019). Consequently, reduced GPR158 levels may aggravate PD pathology by facilitating α-synuclein aggregation (Mallah et al., 2019).

**FIGURE 4:**
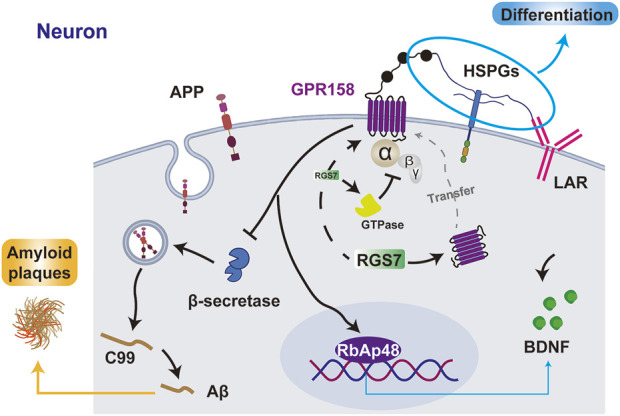
The interaction between GPR158 and related proteins. GPR158 interacts with HSPGs and LAR to promote cell differentiation, inhibits β-secretase to reduce Aβ and amyloid plaque formation, and enhances BDNF expression via RbAp48. Additionally, GPR158 binds RGS7, facilitating its membrane localization and forming a negative feedback loop by promoting GTPase activity. GPR158 also interacts with RGS7 to inhibit the GTPase. APP, amyloid precursor protein; C99, β-CTF fragment of APP.

Therefore, the interaction between GPR158 and related proteins underscores OCN’s potential role in developing NDs (Figure 4).

## 4 Strategies for targeting NDs via OCN/GPR158

Central neuropathies, particularly NDs, present substantial treatment challenges due to two primary factors. First, diagnosis based on behavioral phenotypes is inherently subjective and often delayed. Second, the development of therapeutics for NDs is impeded by limited advancements and significant side effects (Bian et al., 2023). Prior discussions have highlighted the neuronal alterations induced by OCN via GPR158 and their potential mechanisms in developing NDs. Consequently, modulation of OCN and GPR158 may play a pivotal role in influencing both the onset and progression of these diseases. Exercise is valued for its cost-effectiveness and neuroprotective effects. It is increasingly acknowledged as a potential therapeutic approach, partially exerting its effects through the OCN/GPR158 signaling axis.

This section aims to explore the potential of OCN as a disease biomarker and review the impact of exercise on OCN levels, thereby providing a theoretical basis for advancing the diagnosis and treatment of NDs.

### 4.1 OCN/GPR158 as potential risk markers for NDs

A clinical study has demonstrated a correlation between reduced OCN levels, changes in brain microstructure, and cognitive decline (Puig et al., 2016). Runt-related transcription factor 2 (RUNX2), a pivotal transcription factor regulating OCN expression, may exert its effects by directly binding to multiple recognition elements within the OCN promoter and interacting with transcriptional cofactors such as the vitamin D receptor (VDR) to enhance transcriptional activity (Paredes et al., 2004a; Paredes et al., 2004b). Notably, mutations in RUNX2 are linked to cleidocranial dysplasia, a skeletal disorder frequently accompanied by cognitive deficits, suggesting that RUNX2 and its downstream target OCN may have broader roles beyond bone development (Takenouchi et al., 2014). Furthermore, Mendelian randomization established a causal relationship between OCN and various forms of dementia, including AD, PD, Lewy body dementia (LBD), and vascular dementia (VD), with OCN exhibiting a powerful protective effect against AD (Liu et al., 2023). These findings indicate that OCN-related gene expression may be a promising early biomarker for NDs during developmental stages.

The characteristics of GPR158 regarding its brain region and cellular distribution provide a physiological basis for the observed variations in OCN. Overexpression of GPR158 inhibits the proliferation and migration of BTSCs, whereas knockdown of GPR158 enhances these processes (Li et al., 2018). In contrast, GPR158 is highly expressed in oligodendrogliomas and IDH-mutant astrocytomas (Li et al., 2018). Although these studies indicate that GPR158 may exhibit contrasting roles in different types of neurocytomas, either promoting or inhibiting tumor progression, this does not diminish the potential of the OCN/GPR158 axis as a crucial biomarker for diagnosing neurological diseases. On the contrary, it may even enhance its diagnostic sensitivity. Additionally, the post-translational modification profile of GPR158 holds promise as a potential factor associated with diseases, particularly concerning diabetes-related cognitive impairment. As discussed in Section 2.3, chronic hyperglycemia results in increased methylation of GPR158 in the rat hippocampus, adversely affecting learning and memory (Patricia da Silva et al., 2023).

The expression of GPR158 is significantly upregulated in prostate cancer, neuroendocrine tumors of the digestive tract, mucinous ovarian cancer, and various other malignancies (Fu et al., 2022). Moreover, alterations in GPR158 methylation have been observed in esophageal squamous cell carcinoma and melanoma (Oka et al., 2009; Koroknai et al., 2020; Fu et al., 2022), indicating that GPR158 may serve as a potential risk marker beyond NDs.

### 4.2 The impact of exercise on OCN levels

Bone functions as a significant mechanosensitive organ. The presence of mechanosensory resident cells enables mechanical stimulation to trigger metabolic responses in osteoblasts and osteoclasts, thereby promoting bone adaptation to a dynamic environment (Qin et al., 2020). Osteocytes are the primary mechanosensory in bone, capable of detecting fluid shear stress generated by mechanical loading through their extensive dendritic processes (Bonewald, 2011). Mechanical stimulation activates various mechanosensitive structures on the osteocyte membrane, including ion channels such as Piezo1, integrin complexes, and primary cilia (Qin et al., 2020; Li et al., 2025). These activations, in turn, trigger downstream signaling pathways such as Wnt/β-catenin, focal adhesion kinase (FAK), and cyclic AMP (cAMP) signaling (Bonewald and Johnson, 2008; Cuevas et al., 2023; Papaioannou et al., 2024). As described in Section 2.1.3, several of these pathways can directly or indirectly influence the OCN expression in osteoblasts.

After stimulation, bone expresses and secrete a range of osteokines (biologically active molecules secreted by bone tissue with endocrine functions), including OCN, lipocalin-2, sclerostin, Dickkopf-1, and FGF23 (Han et al., 2018). Most osteokines can traverse the blood-brain barrier, establishing the brain as an important target organ (Han et al., 2018) and influencing the development and progression of NDs.

Bone is an integral component of the motor system, constantly subjected to mechanical stress during exercise. As an economical and effective intervention for NDs, the beneficial effects of exercise may be linked to alterations in OCN levels. In recent years, studies have increasingly highlighted the impact of exercise on OCN (Table 2). While the findings are not entirely consistent, several key trends have emerged. First, resistance exercise seems more effective than aerobic exercise in elevating OCN levels during short-term exercise. This phenomenon could be attributed to the more substantial mechanical loading on bone cells during resistance training. Second, serum OCN levels in individuals with obesity appear to be less responsive to exercise, suggesting that individuals with metabolic disorders, such as obesity, may face more significant challenges in deriving benefits from exercise, particularly in terms of OCN regulation.

**TABLE 2:** Effects of exercise on OCN levels.

Exercise duration	Exercise type	Physiological state	OCN level	Reference
Short term	Aerobic exercise	Health	-	Dror et al. (2022)
	Resistance exercise	Health	↑	Koltun et al. (2024)
Long term	Aerobic exercise	Health	↑	Bergquist (1988), Zhang et al. (2020a), Yang et al. (2021), Davidovic Cvetko et al. (2022), Adilakshmi et al. (2024), Hatakeyama et al. (2025)
		Obesity		Jamka et al. (2022)
		Obesity	-	Guzel et al. (2024)
	Resistance exercise/Endurance-strength training/Interval training	Obesity	-	Jamka et al. (2022), Kurgan et al. (2022), Salus et al. (2023)
		Health	↑	Cheng et al. (2020), Honda et al. (2020), Boudenot et al. (2021), Adilakshmi et al. (2024), Hatakeyama et al. (2025)

Currently, research on the effects of exercise on OCN predominantly focuses on serum analyses, with a notable paucity of studies investigating its role in the brain and its relation to GPR158. There is a critical need for rigorous evidence to identify exercise regimens that can effectively optimize OCN/GPR158-mediated pathways to enhance brain health.

## 5 Conclusion and perspective

As a critical receptor for OCN, GPR158 regulates cognitive function by modulating cellular activity, glucose metabolism, synaptic plasticity, and interacting with proteins. However, GPR158 has a dual role in contexts such as tumor development and anxiety/depression. Despite this complexity, it primarily supports cognitive regulation. Additionally, OCN and GPR158 are emerging as potential risk markers for NDs.

The role of GPR158 in the CNS extends beyond its current understanding, particularly in its potential involvement in immune regulation. Mutations in GPR158 have been shown to facilitate the clearance of the hepatitis C virus in patients of European and African descent, thereby reducing the risk of liver damage and related complications (Vergara et al., 2019). Furthermore, single nucleotide polymorphisms (SNPs) in GPR158 are associated with antibody levels in African Americans, and GPR158 (rs12775535) has been identified as a critical candidate gene for immune function (Ovsyannikova et al., 2012). Although the specific mechanisms require further investigation, the insights provided by these studies suggest an additional avenue for enhancing the understanding of the central mechanisms underlying OCN/GPR158.

Future studies on the role of OCN/GPR158 should focus on its multi-ligand and multi-receptor properties. GPR37, another receptor for OCN, is widely expressed in the CNS and shares similarities with GPR158 in regulating neuronal activity. Although GPRC6A is predominantly expressed in peripheral tissues, its connection to metabolic processes offers valuable insights into how GPR158 may regulate cognitive dysfunction linked to glucose metabolism. Thus, when targeting GPR158 for NDs, it is crucial to investigate its interaction with other receptors. Moreover, the ligands of GPR158 are diverse, including glycine, peptides, intracellular binding proteins, steroid hormones, glycosaminoglycans, and miRNA (Lin et al., 2022; Laboute et al., 2023; Rosenkilde and Mathiesen, 2023). This diversity adds complexity to its regulation of cognitive function but may also explain the dual role of GPR158 in different physiological and pathological contexts.

## Glossary

5-HT: Serotonin
AD: Alzheimer’s disease
APs: Action potentials
ASD: Autism spectrum disorders
ATF4: Activating transcription factor 4
AZ: Active zone
Aβ: Amyloid-beta
BDNF: Brain-derived neurotrophic factor
BSP: Bone sialoprotein
BTSCs: Brain tumor stem-like cells
CA3: Cornu ammonis 3
CHOP: C/EBP homologous protein
CNS: Central nervous system
DA: Dopamine
DMP1: Dentin matrix protein 1
ERS: Endoplasmic reticulum stress
FAK: focal adhesion kinase
FGF23: Fibroblast growth factor 23
GAD1/2: Glutamate decarboxylase 1/2
GPR158: G protein-coupled receptor 158
HD: Huntington’s disease
HMGB1: High mobility group box-1 protein
HSPA5: Heat shock protein family A (Hsp70) member 5
HSPGs: Heparan sulfate proteoglycans
LAR: Leukocyte common antigen-related
LBD: Lewy body dementia
LRP5/6: Low-density lipoprotein receptor-related proteins 5 and 6
LTD: Long-term depression
LTP: Long-term potentiation
MF: Mossy fiber
MSNs: Medium spiny neurons
NDs: Neurodegenerative diseases
NE: Norepinephrine
NGF: Nerve growth factor
NLS: Nuclear localization signal
OCN: Osteocalcin
OPG: Osteoprotegerin
OPN: Osteopontin
PD: Parkinson’s disease
PPF: Paired-pulse facilitation
RANKL: Receptor activator of nuclear factor-κB ligand
RGS7: Regulator of G protein signaling 7
sEPSCs: Spontaneous excitatory postsynaptic currents
SIBLING: Small integrin-binding ligand N-linked glycoprotein
SMA: Spinal muscular atrophy
Snap25: Synaptosome associated protein 25
SNARE: Soluble N-ethylmaleimide-sensitive factor attachment protein receptors
TH: Tyrosine hydroxylase
TP53: Tumor protein 53
TPH2: Tryptophan hydroxylase 2
UPR: Unfolded protein response
VD: Vascular dementia
VDR: Vitamin D receptor
WT: Wild-type
XBP1: X-box binding protein 1
